# Use of partitioned GMM marginal regression model with time-dependent covariates: analysis of Chinese Longitudinal Healthy Longevity Study

**DOI:** 10.1186/s12874-020-01003-0

**Published:** 2020-05-24

**Authors:** Elsa Vazquez-Arreola, Dan Xue, Jeffrey R. Wilson

**Affiliations:** 1 School of Mathematical and Statistical Sciences, Arizona State University, Tempe, AZ 85287 USA; 2 Department of Economic, W.P. Carey School of Business, Arizona State University, Tempe, AZ 85287 USA

**Keywords:** Correlated data, Generalized linear models, Partitioned coefficients

## Abstract

**Background:**

Elderly population’s health is a major concern for most industrial nations. National health surveys provide a measure of the state of elderly health. One such survey is the Chinese Longitudinal Healthy Longevity Survey. It collects data on risk factors and outcomes on the elderly. We examine these longitudinal survey data to determine the changes in health and to identify risk factors as they impact health outcomes including the elderly’s ability to do a physical check.

**Methods:**

We use a Partitioned GMM logistic regression model to identify risk factors. The model also accounts for the correlation between lagged time-dependent covariates and the outcomes. It addresses present and past measures of time-dependent covariates on simultaneous outcomes. The relation produces additional regression coefficients as byproduct of the Partitioned model, identifying the immediate, delayed effects (lag − 1), further delayed (lag-2), etc. Therefore, the model presents the opportunity for decision makers to monitor the covariate over time. This technique is particularly useful in healthcare and health related research. We use the Chinese Longitudinal Health Longevity Survey data to identify those risk factors and to display the utility of the model.

**Results:**

We found that one’s ability to make own decisions, frequently consuming vegetables, exercise frequently, one’s ability to transfer without assistance, having visual difficulties and being able to pick book from floor while standing had varying effects of significance on one’s health and ability to complete physical checks as they get older.

**Conclusions:**

The partitioning of the covariates as immediate effect, delayed effect or further delayed effect are important measures in a declining population.

## Background

Longitudinal studies in medical-related research are useful in identifying changes in outcomes as impacted by certain risk factors. While the repeated measurements on subjects generate correlated observations, they are of different types of correlation. There is correlation among the responses. There is correlation between the time-dependent covariates and the response. These correlations have different impacts on the outcomes. Thus, any models fitted to these data need to address these correlations accordingly.

Modelling time-dependent covariates when analyzing binary outcomes in longitudinal studies has drawn attention. There are methods due to Generalized Estimating Equations (GEE) and others based on Generalized Method of Moments (GMM) [1–5]. However, these methods do not separate out the impact of the time-dependent covariates on the outcomes. In fact, they provide estimates that represent an average of the impacts. Obermeier et.al [6]. suggested that when modeling longitudinal data, one could not assume that the association between a time-dependent covariate and the outcome was only direct and simultaneous. This is because the outcome might depend on past measurements of the covariate. Thus, an alternative approach is to separate the different impacts of the covariate. Heagerty [7] indicated that one way to properly model longitudinal outcomes with time-dependent covariates is to include appropriate lagged values of such covariates. This approach requires additional regression coefficients for each segment of time-dependent covariate. These additional coefficients allow parsing of the effect of the covariate on the response, rather than assuming that the association maintains the same strength and direction over time. It provides insight into the effects of time-dependent covariates on present and future values of the outcomes.

### Motivating example

Elderly population’s health is a major concern for most industrial nations. National health surveys provide a measure of the state of elderly health. One such survey uses the Chinese Longitudinal Healthy Longevity Study (CLHLS) [8]. It collects data on risk factors and outcomes on the elderly population. The CLHLS was designed to identify key factors contributing to healthy longevity among elderly adults in China. The survey was conducted over time but we concentrated on four waves 2005, 2008, 2011 and 2014. This survey is of particular interest in China, as their annual growth rate of the elderly population is approximately 4.4% and approximately 20% of the world’s oldest population live in China [8]. Gu, Zhang and Zeng [9] investigated the impact of adequate access to healthcare. Li, Zhang and Liang [10] used waves 1 & 2 to determine how living arrangements in 1998 impacted self-rated health in 2000. Zheng et.al [11]. studied the associations of environmental variables. Wu and Schimmele [12] tested how levels of psychological disposition in 1998 impacted self-rated health in 2000. Wang, Zheng, Kurosawa and Inaba [13] studied gender and age differences in health among elderly Chinese using data collected in 2002. However, in all of these studies only one or two waves of data were used and researchers were only able to determine cross-sectional or lag-1 effects of time-dependent covariates on the outcomes.

In this paper, we made use of four waves to demonstrate the fit of Partitioned GMM for binary simultaneous outcomes, completion of a physical check and their health status. These responses were objectively measured by an interviewer. There are subjective measures but we concentrated on the objective measures. We focused our attention on the longitudinal aspect of the data and used all four waves. This increased number of waves used allows us to optimize the longitudinal nature of the data.

### Data

The data consisted of elderly people 64 years and older living in 22 of 31 provinces in China. There were 8084 observations measured on 2021 individuals over the four waves. We fit models to interviewer-rated health and completion of a physical check that included the time-independent covariate gender. These models also included the time-dependent covariates: able to make own decision, consumed vegetables frequently, exercised, transfer without assistance, visual difficulty and ability to pick up book from floor while standing. Descriptive statistics for the outcomes and time-dependent covariates are given in Tables 1 and 2, respectively. Our initial observation suggested a steady decline in the percentage of interviewees considered healthy over time, Table 1.

**Table 1:** Descriptive Statistics for four outcomes (%)

Outcomes	Year 2005	Year 2008	Year 2011	Year 2014
Interviewer-rated health	94.76	94.41	91.98	86.69
Complete physical check	97.48	94.66	95.84	93.42

**Table 2:** Descriptive Statistics for time-dependent covariates (%)

C**ovariate**	Y**ear** 2005	Y**ear** 2008	Y**ear** 2011	Y**ear** 2014
Make own decisions	90.20	87.38	89.36	87.33
Consume vegetables frequently	89.56	91.69	92.53	90.95
Exercise	38.05	42.70	48.79	39.53
Transfer without assistance	99.70	99.46	98.17	95.10
Visual difficulties	19.40	19.54	23.80	29.54
Pick up book from floor while standing	91.98	91.64	85.85	77.04

## Methods

**Fig. 1:**
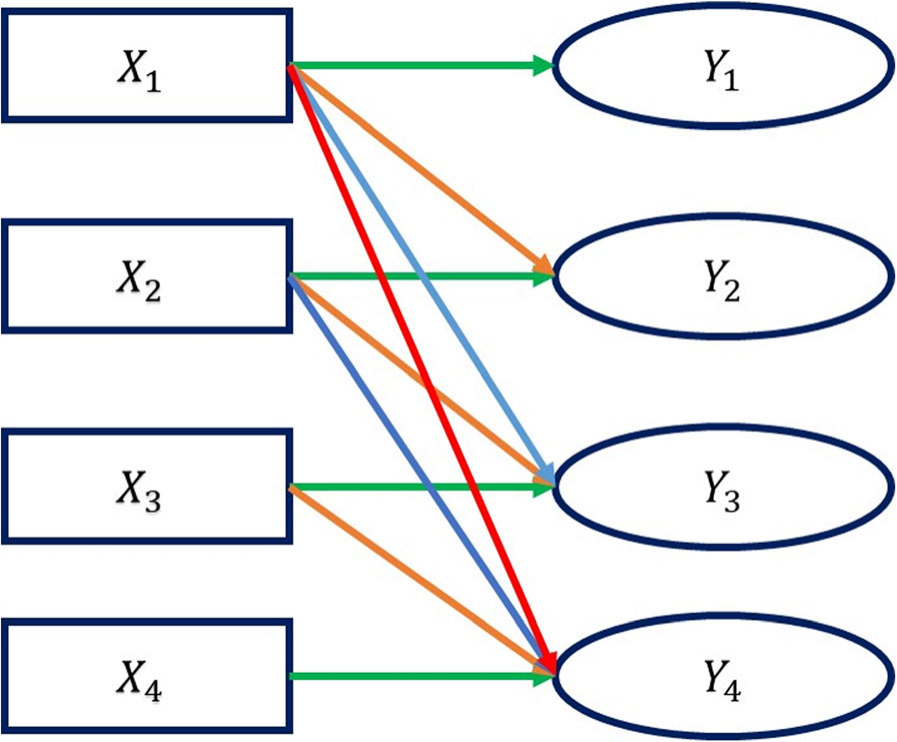
Relationship between time-dependent covariate ***X*** and ***Y*** across four time-periods

We fit a partitioned GMM logistic regression model [14] to the Chinese Longitudinal Healthy Longevity Study data to determine the effects of time-dependent covariates on the binary outcomes. The model measures the impact of time independent and time-dependent covariates ***X*** on the outcome *Y* measured at four different time points. Thus, there are some relations between ***X*** and *Y* other than cross sectional that must be addressed, Fig. 1. Thus, the partitioned GMM logistic regression model [14] provides coefficient estimates for the effect of ***X*** on *Y* when both are measured at the same time, for when ***X*** is measured one-time period ahead of *Y*, for when ***X*** is measured two-time periods ahead to *Y* and for when ***X*** is measured three-time periods ahead to *Y*.

### Partitioned GMM logistic regression models with time dependent covariates

Let y_it_ denote the binary observation for individual *i* (*i* = 1, …, *N*) at time *t* (*t* = 1, …, *T*). Let ***x***_*it*_ = (*x*_*i*1*t*_, …, *x*_*iJt*_) be a vector of *J* time-dependent covariates, where *x*_*ijt*_ is the *j*^*th*^ covariate observed at time *t* for individual *i*. Assume that observations *y*_*is*_ and *y*_*kt*_ are independent when *i* ≠ *k* but not necessarily when *i* = *k* and *s* ≠ *t*. The Partitioned GMM logistic regression model accounts for the relationships between the outcomes observed at time *t*, ***y***_*i*_ = (*y*_*i*1_, .., *y*_*iT*_) and the *j*^*th*^ covariate observed at time *s*, *x*_*ijs*_ for *s* ≤ *t*. For each subject *i* and each time-dependent covariate *x*_*ijt*_ measured at times *t* = 1, 2, …, *T*; the data matrix is reconfigured as a lower triangular matrix,

$$ {\boldsymbol{X}}_{ij}=\left[\begin{array}{c}{x}_{ij1}\\ {}{x}_{ij2}\\ {}\begin{array}{c}\vdots \\ {}{x}_{ij T}\end{array}\end{array}\ \begin{array}{ccc}0& \dots & 0\\ {}{x}_{ij1}& \dots & 0\\ {}\begin{array}{c}\vdots \\ {}{x}_{ij\left(T-1\right)}\end{array}& \begin{array}{c}\vdots \\ {}\dots \end{array}& \begin{array}{c}\vdots \\ {}{x}_{ij1}\end{array}\end{array}\right]=\left[{\boldsymbol{x}}_{ij}^{\left[0\right]}\kern0.5em {\boldsymbol{x}}_{ij}^{\left[1\right]}\kern0.5em \dots \kern0.5em {\boldsymbol{x}}_{ij}^{\left[T-1\right]}\right] $$

where the superscript denotes the difference, *t* − *s* in time-periods between the response time *t* and the covariate time *s*. In this matrix, ${\boldsymbol{x}}_{ij}^{\left[0\right]}$ contains values of the time-dependent covariate observed at the same time as the outcome, ${\boldsymbol{x}}_{ij}^{\left[1\right]}$ includes values of the time-dependent covariate observed one-time period prior to outcomes, and so on such that ${\boldsymbol{x}}_{ij}^{\left[T-1\right]}$ consists of the values of the covariate measured *T* − 1 time periods prior to outcome. Thus, the model for the outcome at time *t* with one time-independent covariate and one time-dependent covariate is

1 $$ logit\left({\mu}_{it}\right)={\beta}_0+{\beta}_F{x}_F+{\beta}_j^{tt}{x}_{ij t}+{\beta}_j^{\left[1\right]}{x}_{ij\left(t-1\right)}+{\beta}_j^{\left[2\right]}{x}_{ij\left(t-2\right)}+\dots +{\beta}_j^{\left[t-1\right]}{x}_{ij1} $$

while the model for all time periods in matrix form is

$$ logit\left[\begin{array}{c}{\mu}_{i1}\\ {}{\mu}_{i2}\\ {}\begin{array}{c}\vdots \\ {}{\mu}_{iT}\end{array}\end{array}\right]={\beta}_0\left[\begin{array}{c}1\\ {}1\\ {}\begin{array}{c}\vdots \\ {}1\end{array}\end{array}\right]+{\beta}_F{x}_F\left[\begin{array}{c}1\\ {}1\\ {}\begin{array}{c}\vdots \\ {}1\end{array}\end{array}\right]+{\beta}_j^{tt}{\boldsymbol{x}}_{ij}^{\left[0\right]}+{\beta}_j^{\left[1\right]}{\boldsymbol{x}}_{ij}^{\left[1\right]}+{\beta}_j^{\left[2\right]}{\boldsymbol{x}}_{ij}^{\left[2\right]}+\dots +{\beta}_j^{\left[T-1\right]}{\boldsymbol{x}}_{ij}^{\left[T-1\right]} $$

The coefficient ${\beta}_j^{tt}$ denotes the effect of the covariate *x*_*ijt*_ on the response *Y*_*t*_ when both are observed in the same time-period, while the vector of coefficient *β*_*F*_ denotes the effect of the time-independent covariate *x*_*F*_ on the response *Y*_*t*_. When *s* < *t*, we denote the lagged effect of the covariate *x*_*js*_ on the response *Y*_*t*_ by the coefficients ${\beta}_j^{\left[1\right]},{\beta}_j^{\left[2\right]},\dots, {\beta}_j^{\left[T-1\right]}$. In general, each of the *J* time-dependent covariates yield a maximum of *T* partitions of *β*_*j*_. Thus, for a model with *J* covariates, the data matrix ***X*** has a maximum dimension of *NT* by (*J* × *T*) + 1, and ***β*** is a vector of maximum length (*J* × *T*) + 1.

This method of estimating regression coefficients relies on valid moment conditions resulting from the covariate values at different times on the outcome at other times. The moment conditions are valid at cross-sectional measurements where covariates are measured at the same time as the outcome [2]. However, valid moment conditions between lagged covariates and the outcomes need to be tested. We do so through a test of bivariate correlation developed by Lalonde, Wilson and Yin [3]. Once the valid moments are identified, the regression parameters are estimated using a GMM approach [14]. We do not rehash the derivations here. We encourage the readers, who want to see that development to go to Lalonde, Wilson, and Yin [3], and Irimata, Broatch, and Wilson [14]. We fit these models through SAS 9.4 software using the %partitionedGMM macro (https://github.com/kirimata/Partitioned-GMM) [15]. It includes the test for valid moment conditions [3].

In our analysis of data in CLHLS, we fit two partitioned GMM logistic regression models to model interviewer-rated health and interviewees’ ability to complete a physical check separately.

## Results

### Health

Immediate impacts were identified for vegetables (OR = 1.70 with 95% CI: 1.30, 2.23), exercise (OR = 2.03 with 95% CI: 1.52, 2.71), transfer without assistance (OR = 3.65 with 95% CI: 2.39, 5.59), having visual difficulties (OR = 0.64 with 95% CI: 0.49, 0.84) and pick book from floor while standing (OR = 4.11 with 95% CI: 3.11, 5.43), Table 3. For a one time-period lag (i.e. delayed effect), exercise (OR = 1.39 with 95% CI: 1.03, 1.89) and transfer without assistance (OR = 1.76 with 95% CI: 1.05, 2.95) significantly impacts the outcome. Across a two-time period lag (further delayed effect), transfer without assistance (OR = 0.44 with 95% CI: 0.24, 0.81) had a significant impact on this outcome, Table 3. There were no significant effects across a three-time period lag (furthermost delayed effect).

**Table 3:** Results of partitioned GMM model for interviewer-rated health and ability to complete physical check

Time period	Covariate	Interviewer-rated health			Physical check		
		OR	OR (95% CI)	*p*-value	OR	OR (95% CI)	*p*-value
Cross sectional	Male	0.96	[0.74, 1.25]	0.77	1.34	[0.90, 1.99]	0.15
	Own decision	1.27	[0.95, 1.71]	0.11	1.61	[1.05, 2.48]	0.03
	Vegetables	1.70	[1.30, 2.23]	< 0.001	0.80	[0.48, 1.35]	0.40
	Exercise	2.03	[1.52, 2.71]	< 0.001	1.74	[0.94, 3.24]	0.08
	Ability to transfer	3.65	[2.39, 5.59]	< 0.001	13.83	[8.23, 23.27]	< 0.001
	Visual difficulties	0.64	[0.49, 0.84]	< 0.001	0.39	[0.25, 0.61]	< 0.001
	Pick book standing	4.11	[3.11, 5.43]	< 0.001	5.88	[4.04, 8.54]	< 0.001
One time period lag	Own decision	0.96	[0.65, 1.44]	0.85	0.54	[0.23, 1.25]	0.15
	Vegetables	0.77	[0.51, 1.15]	0.20	1.29	[0.69, 2.38]	0.42
	Exercise	1.39	[1.03, 1.89]	0.03	0.88	[0.59, 1.33]	0.54
	Ability to transfer	1.76	[1.05, 2.95]	0.03	0.64	[0.23, 1.75]	0.38
	Visual difficulties	1.05	[0.78, 1.41]	0.75	0.73	[0.47, 1.15]	0.17
	Pick book standing	0.75	[0.51, 1.11]	0.16	1.37	[0.91, 2.07]	0.13
Two time period lag	Own decision	0.90	[0.61, 1.32]	0.58	0.67	[0.29, 1.57]	0.36
	Vegetables	1.12	[0.74, 1.70]	0.60	0.53	[0.27, 1.02]	0.06
	Exercise	1.03	[0.77, 1.39]	0.83	0.94	[0.52, 1.71]	0.84
	Ability to transfer	0.44	[0.24, 0.81]	0.01	4.30	[1.78, 10.43]	< 0.001
	Visual difficulties	1.10	[0.77, 1.57]	0.60	1.47	[0.75, 2.88]	0.26
	Pick book standing	1.43	[0.90, 2.26]	0.13	1.18	[0.55, 2.53]	0.68
Three time period lag	Own decision	–	–		0.31	[0.09, 1.14]	0.08
	Vegetables	1.32	[0.87, 2.01]	0.19	2.12	[1.04, 4.33]	0.04
	Exercise	0.93	[0.63, 1.37]	0.71	1.34	[0.60, 2.98]	0.48
	Ability to transfer	0.67	[0.36, 1.25]	0.21	1.42	[0.33, 6.01]	0.64
	Visual difficulties	0.73	[0.50, 1.06]	0.10	2.01	[0.82, 4.97]	0.13
	Pick book standing	0.90	[0.52, 1.56]	0.72	–	–	–

### Complete physical check

Immediate impacts were obtained for making own decisions (OR = 1.61 with 95% CI: 1.05, 2.48), transfer without assistance (OR = 13.83 with 95% CI: 8.23, 23.27), visual difficulties (OR = 0.39 with 95% CI: 0.25, 0.61) and pick up book from floor while standing (OR = 5.88 with 95% CI: 4.04, 8.54), Table 3, Fig. 3.

Further impacts were seen at lag-2 for transfer without assistance (OR = 4.30 with 95% CI: 1.78, 10.43). An additional delayed impact at lag-3 was seen for eating vegetables frequently (OR = 2.12 95% CI: 1.04, 4.33), Table 3, Fig. 3.

## Discussion

The uniqueness of the partitioned GMM logistic regression models allows the immediate effect as well as future effects of time-dependent covariates on outcomes to be measured. Unlike the previous studies, researchers analyzed the CLHLS data but were only able to estimate cross-sectional or lag-1 effects of time-dependent covariates. However, we were able to determine both cross-sectional and lag-1 associations as well as lag-2 and lag-3 relationships between the time-dependent covariates and our two binary outcomes, Table 4.

**Table 4:** Positive and negative effects over time of time-dependent covariates on interviewer-rated health and physical check completion

	Interviewer rated health				Physical check			
Covariate	Cross-sectional	One time period lag	Two time period lag	Three time period lag	Cross-sectional	One time period lag	Two time period lag	Three time period lag
Own decision					+			
Vegetables	+							+
Exercise	+	+						
Ability to transfer	+	+	–		+		+	
Visual difficulties	–				–			
Pick book standing	+				+			

**Fig. 2:**
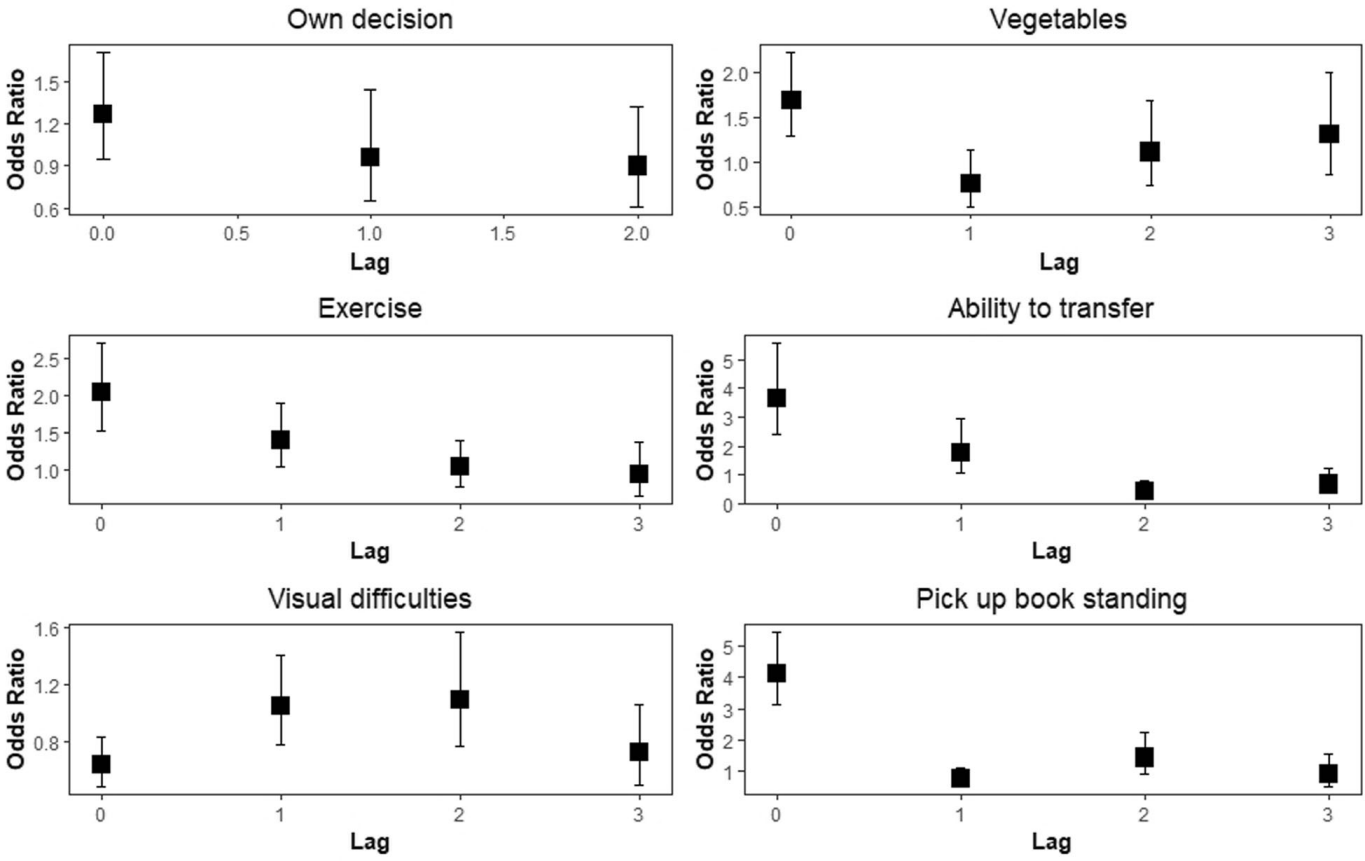
Partitioned GMM model for interviewer-rated health (odds ratio estimates and 95% confidence intervals)

Figure 2 presents the relationships between the time-dependent covariates and interviewer-rated health, over time. We found that gender and the ability to make one’s own decision did not impact the probability of good health. Frequent consumption of vegetables increased good health immediately, but did not have any significant lagged effects. Exercising significantly increased the likelihood of being in good health immediately and in the next time period. The ability to transfer without assistance has a positive impact on good health immediately and in the next time period. Having visual challenges has an immediate negative impact on having good health. The ability to pick book from floor while standing has an immediate positive impact on good health.

**Fig. 3:**
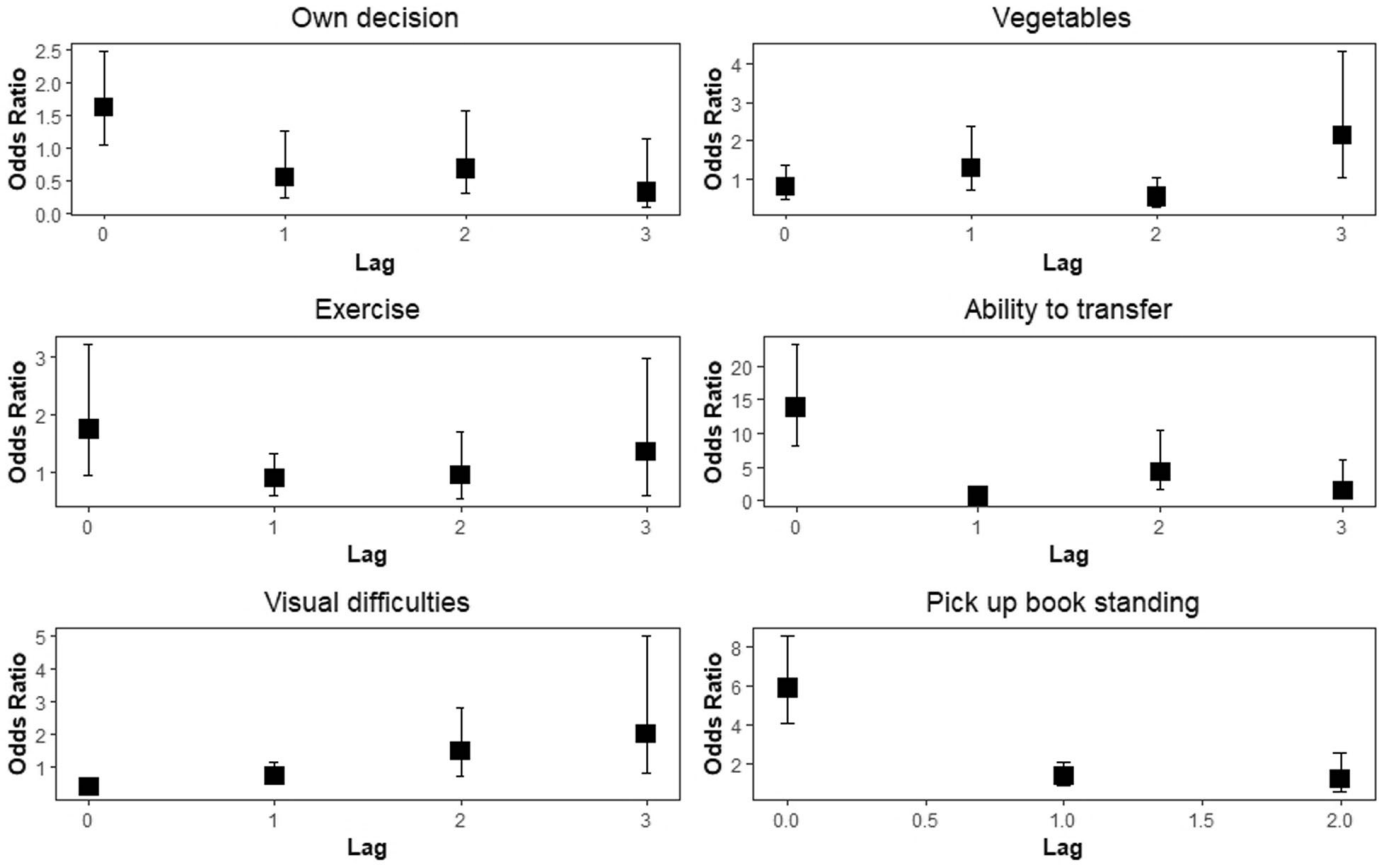
Partitioned GMM model for ability to complete physical check (odds ratio estimates and 95% confidence intervals)

Gender did not significantly impact the likelihood of completing a physical check. The ability to make one’s own decisions has an immediate positive impact on completing a physical check. Consumption of vegetables frequently in the first wave significantly increased the likelihood of completing a physical check in the last wave. Exercising did not impact the completion of a physical check at any point in time. Ability to transfer without assistance significantly increases the likelihood of completing a physical check immediately and across a two time-period lag. Having visual challenges negatively impacted completing a physical check immediately. Being able to pick up a book from floor while standing increases the probability of completing a physical check. Figure 3 presents the changing relationships between the time-dependent covariates and the ability to complete a physical check.

## Conclusions

Though we fitted the Partitioned GMM model to two binary outcomes, this model readily accommodates continuous outcomes. The partitioning of the data matrix with the use of additional coefficients provides an opportunity to measure the covariate on the responses at different periods.

## Data Availability

The dataset analyzed during the current study is available at the Inter-University Consortium for Political and Social Research repository, https://www.icpsr.umich.edu/icpsrweb/ICPSR/studies/36692.

## Abbreviations

CLHLS: Chinese Longitudinal Healthy Longevity Study
GMM: Generalized Method of Moments
WPA: World Population Aging
OR: Odds Ratio
CI: Confidence Interval
